# Modulation of type 1 cannabinoid receptor activity by cannabinoid by-products from *Cannabis sativa* and non-cannabis phytomolecules

**DOI:** 10.3389/fphar.2022.956030

**Published:** 2022-08-26

**Authors:** Ayat Zagzoog, Ashley Cabecinha, Hanan Abramovici, Robert B. Laprairie

**Affiliations:** ^1^ College of Pharmacy and Nutrition, University of Saskatchewan, Saskatoon, SK, Canada; ^2^ Office of Cannabis Science and Surveillance, Controlled Substances and Cannabis Branch, Health Canada, Ottawa, ON, Canada; ^3^ Department of Pharmacology, College of Medicine, Dalhousie University, Halifax, NS, Canada

**Keywords:** cannabinoid, phytomolecule, terpene, type 1 cannabinoid receptor, molecular pharmacology, cAMP, βarrestin

## Abstract

*Cannabis sativa* contains more than 120 cannabinoids and 400 terpene compounds (i.e., phytomolecules) present in varying amounts. *Cannabis* is increasingly available for legal medicinal and non-medicinal use globally, and with increased access comes the need for a more comprehensive understanding of the pharmacology of phytomolecules. The main transducer of the intoxicating effects of *Cannabis* is the type 1 cannabinoid receptor (CB1R). ∆^9^-tetrahydrocannabinolic acid (∆^9^-THCa) is often the most abundant cannabinoid present in many cultivars of *Cannabis*. Decarboxylation converts ∆^9^-THCa to ∆^9^-THC, which is a CB1R partial agonist*.* Understanding the complex interplay of phytomolecules—often referred to as “the entourage effect”—has become a recent and major line of inquiry in cannabinoid research. Additionally, this interest is extending to other non-*Cannabis* phytomolecules, as the diversity of available *Cannabis* products grows. Here, we chose to focus on whether 10 phytomolecules (∆^8^-THC, ∆^6a,10a^-THC, 11-OH-∆^9^-THC, cannabinol, curcumin, epigallocatechin gallate, olivetol, palmitoylethanolamide, piperine, and quercetin) alter CB1R-dependent signaling with or without a co-treatment of ∆^9^-THC. Phytomolecules were screened for their binding to CB1R, inhibition of forskolin-stimulated cAMP accumulation, and βarrestin2 recruitment in Chinese hamster ovary cells stably expressing human CB1R. Select compounds were assessed further for cataleptic, hypothermic, and anti-nociceptive effects on male mice. Our data revealed partial agonist activity for the cannabinoids tested, as well as modulation of ∆^9^-THC-dependent binding and signaling properties of phytomolecules *in vitro* and *in vivo*. These data represent a first step in understanding the complex pharmacology of *Cannabis*- and non-*Cannabis-*derived phytomolecules at CB1R and determining whether these interactions may affect the physiological outcomes, adverse effects, and abuse liabilities associated with the use of these compounds.

## Introduction

**FIGURE 1:**
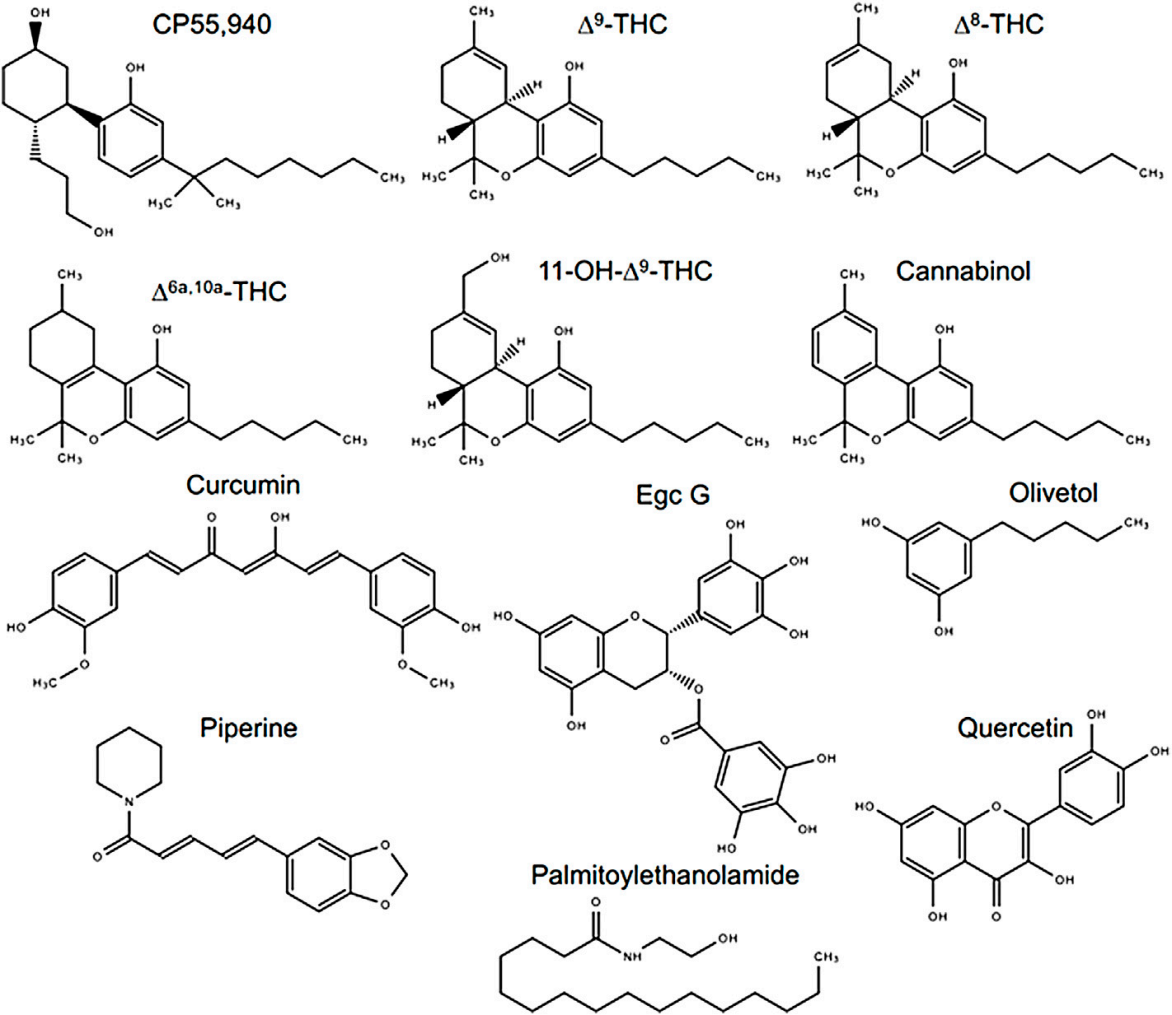
Compounds assessed in this study. Chemical structures were drawn in Microsoft PowerPoint by the authors. Egc G, epigallocatechin gallate.

In 2018, Canada became the first G7 country to legalize *Cannabis sativa* for non-medical purposes. Despite decades of research and significant legislative and policy advances, our scientific understanding of *Cannabis* and cannabinoid pharmacology remains quite limited. Gaining a more comprehensive understanding of cannabinoid pharmacology to better shed further light on the beneficial and harmful effects of cannabis is critically important. There are many biologically active compounds in cannabis. “Phytocannabinoids” are generally 21-carbon bicyclic compounds, some of which are known to act at the cannabinoid receptors. Terpenes, on the other hand, are a large and structurally diverse group of hydrocarbon molecules present in nearly all plants that produce characteristic odors. Here, we will refer to these groups collectively as “phytomolecules.” The two best known phytocannabinoids are ∆^9^-tetrahydrocannabinol (∆^9^-THC) and cannabidiol (CBD). All vertebrates studied to date also naturally produce endogenous cannabinoids anandamide (AEA) and 2-arachidonoylglycerol (2-AG). These cannabinoids act to modulate the brain and body’s cannabinoid receptors: CB1R and CB2R, which act to limit neurotransmitter release throughout the brain and inflammatory processes, respectively (Pertwee, 2008). Beyond ∆^9^-THC and CBD, more than 500 phytomolecules have been identified in extracts from the *Cannabis* plant (Figure 1) (Howlett and Abood, 2017). The pharmacodynamic and pharmacokinetic properties of these compounds alone and in unique combinations present in plant chemotypes remain largely unknown (Pertwee, 2008; Howlett and Abood, 2017).

In Canada, ∆^9^-THC and CBD content in legal *Cannabis* products is monitored and must be labeled on *Cannabis* products for retail sale because these constituents are recognized as being pharmacologically active drug compounds, and in the case of ∆^9^-THC, are known to produce intoxication and other effects. However, it is possible that minor constituents of *Cannabis* products may also be psychoactive and/or intoxicating, and it is important to explore that aspect further to better understand the risks of cannabis use. The cannabis product market is also rapidly evolving. Products such as cannabis extracts, cannabis topicals, and edible cannabis often contain ingredients in addition to cannabis. As the variety of cannabis products increases, so does the landscape of ingredients that are being or could be combined with cannabis. It is unknown whether some of the ingredients used in cannabis products, or components thereof, activate or modulate cannabinoid receptor activity. While the majority of ingredients are likely to be benign, it is important to understand those that may impact cannabinoid receptor activity and impact the risk profile of such products. Furthermore, phytomolecules may activate or modulate cannabinoid receptor activity via inhibitory, agonist, partial agonist, or allosteric mechanisms. This novel pharmacology would increase our knowledge about what types of substances change cannabinoid receptor activity—and how—which we can then correlate to their chemical structures. Knowledge of the structure–activity relationship between a substance’s structure and its function at a receptor can lead to the creation of a “novel drug scaffold;” that is, a bare minimum chemical structure required to produce some known biological effect. Therefore, the research undertaken here could yield novel drug scaffolds based on phytomolecules for new—previously unknown—therapies.

The potential unique pharmacological effects arising through unique combinations of *Cannabis* phytomolecules are referred to as “the entourage effect” (Russo 2011). Several *in vitro* studies have shown that some terpenes present in *Cannabis*—α- and β-pinene, β-caryophyllene, β-myrcene, linalool, α-humulene, and limonene—do not have direct modulatory effects on CB1R, CB2R, transient receptor potential ankyrin 1 (TRPA1), or transient receptor potential vanilloid 1 (TRPV1) (Santiago et al., 2019; Finlay et al., 2020; Heblinski et al., 2020). Our group recently demonstrated that select, isolated, minor cannabinoids display weak partial agonist activity on CB1R and CB2R (Zagzoog et al., 2020). Also, there is mounting evidence from rodent studies that *Cannabis* products produce different pharmacology from isolated ∆^9^-THC or CBD (Devsi et al., 2020; LaVigne et al., 2021; Roebuck et al., 2022). In addition, our group and several others have shown that cannabinoids may behave as allosteric ligands in cell culture models, modulating the signaling of GPCRs via indirect means (Laprairie et al., 2015; Martinez-Pinilla et al., 2017; Tham et al., 2018; Navarro et al., 2021). Consequently, cannabinoids are phytomolecules likely capable of affecting cellular signaling through a myriad of mechanisms. It is important to continue assessing combinations of phytomolecules to determine how these mixtures may alter the pharmacodynamics and pharmacokinetics of *Cannabis* products.

CB1R is activated by a wide spectrum of structurally diverse molecules (Howlett and Abood, 2017). CB1R modulates intracellular signaling through Gα_i/o_-dependent inhibition of cAMP, inhibition of Ca^2+^ currents and inwardly rectifying K^+^ channels, and recruitment of βarrestins. Therefore, in order to understand the potential effects that phytomolecules may have on CB1R, it is important to assess several endpoints, such as ligand binding, G-protein-dependent signaling, and βarrestin recruitment.

The purpose of this study was to assess 10 select phytomolecules (∆^8^-THC, ∆^6a,10a^-THC, 11-OH-∆^9^-THC, cannabinol [CBN], curcumin, epigallocatechin gallate [Egc G], olivetol, palmitoylethanolamide [PEA], piperine, and quercetin) in combination with ∆^9^-THC to determine how these compounds modulate ligand binding and signaling *via* CB1R *in vitro*, and catalepsy, body temperature, and nociception *in vivo*. These data represent an initial step in determining whether phytomolecules could alter the pharmacological effects of cannabinoids and cannabis.

## Materials and methods

### Compounds

Compounds were purchased from Sigma-Aldrich (Oakville, ON), with the exceptions of ∆^9^-THC, which was purchased from Toronto Research Chemicals (Toronto, ON) and SR141716A, which was purchased from Cayman Chemicals (Ann Arbor, MI). [^3^H]CP55,940 (174.6 Ci/mmol) was obtained from PerkinElmer (Guelph, ON). All reagents were obtained from Sigma-Aldrich unless specifically noted. Compounds were dissolved in DMSO (final concentration of 0.1% in assay media for all assays) and added directly to the media at the concentrations and times indicated. For all experiments, 0.1% DMSO was used as the vehicle control.

### Cell culture

Chinese hamster ovary (CHO)-K1 cells stably expressing human cannabinoid CB1R (hCB1R) were maintained at 37°C and 5% CO_2_ in F-12 DMEM containing 1 mM L-glutamine, 10% FBS, and 1% penicillin–streptomycin as well as hygromycin B (300 μg/ml) and G418 (600 μg/ml) (Bolognini et al., 2012). For membrane preparation, cells were removed from flasks by scraping, centrifuged, and then frozen as a pellet at −80°C until required. Before use in a radioligand binding assay, cells were defrosted, diluted in Tris buffer (50 mM Tris–HCl and 50 mM Tris–base), and homogenized with a 1 ml hand-held homogenizer (Bolognini et al., 2012). HitHunter (cAMP) and PathHunter (βarrestin2) CHO-K1 cells stably expressing hCB1R from DiscoveRx^®^ (Eurofins, Fremont, CA) were maintained at 37°C and 5% CO_2_ in F-12 DMEM containing 10% FBS and 1% penicillin–streptomycin with 800 μg/ml geneticin (HitHunter) or 800 μg/ml G418 and 300 μg/ml hygromycin B (PathHunter).

### CHO cell membrane preparation and radioligand displacement assay

As described in previous work from our group, CHO-K1 hCB1R cells were disrupted by cavitation in a pressure cell and membranes were sedimented by ultracentrifugation (Zagzoog et al., 2020). The pellet was resuspended in TME buffer (50 mM Tris–HCl, 5 mM MgCl_2_, and 1 mM EDTA, pH 7.4), and membrane proteins were quantified with a Pierce BCA Protein Assay Kit (Thermo Scientific, Rockford, United States).

Radioligand binding assays were carried out as described previously (Zagzoog et al., 2020). Briefly, binding was initiated by mixing CHO-K1 hCB1R cell membranes (25 μg protein per well) with 1 nM [^3^H]CP55,940 in Tris binding buffer (50 mM Tris–HCl, 50 mM Tris–base, and 0.1% BSA, pH 7.4; total assay volume 2 ml), followed immediately by the addition of the compounds or vehicle (0.1% DMSO). All assays were performed at 37°C for 120 min before termination by the addition of ice-cold Tris-binding buffer, followed by vacuum filtration using a 24-well sampling manifold (Brandel Cell Harvester; Brandel Inc., Gaithersburg, MD, United States). Each reaction well was washed six times with a 1.2 ml aliquot of Tris-binding buffer. The filters were air-dried and then placed in 5 ml of scintillation fluid overnight (Ultima Gold XR, PerkinElmer). Radioactivity was quantified by liquid scintillation spectrometry. Specific binding was calculated as the difference between the binding that occurred in the presence and absence of 1 μM unlabeled CP55,940.

### HitHunter cAMP assay

Inhibition of forskolin (FSK)-stimulated cAMP was determined using the DiscoveRx HitHunter assay in CHO-K1 hCB1R cells as described previously (Zagzoog et al., 2020). A total of 20,000 CHO-K1 hCB1R cells/well were grown in low-volume 96-well plates and incubated overnight in Opti-MEM containing 1% FBS at 37°C and 5% CO_2_. Opti-MEM was removed and replaced with cell assay buffer (DiscoveRx), and the cells were simultaneously treated at 37°C with 10 μM FSK and compounds for 90 min. cAMP antibody solution and cAMP working detection solutions were added according to the manufacturer’s directions (DiscoveRx), and cells were incubated for 60 min at room temperature. cAMP solution A was added (DiscoveRx), and cells were incubated for an additional 60 min at room temperature before chemiluminescence was measured on a Cytation5 plate reader (top read, gain 200, integration time 10,000 ms).

### PathHunter βarrestin2 assay

βarrestin2 recruitment was measured as described previously (Zagzoog et al., 2020). A total of 20,000 CHO-K1 hCB1R cells/well were grown in low-volume 96-well plates and incubated overnight in Opti-MEM containing 1% FBS at 37°C and 5% CO_2_. Cells were simultaneously treated at 37°C with compounds for 90 min. A detection solution was added to the cells according to the manufacturer’s directions (DiscoveRx), and the cells were incubated for 60 min at room temperature. Chemiluminescence was measured on a Cytation5 plate reader (top read, gain 200, integration time 10,000 ms).

### *In vivo* analyses

Male C57BL/6 mice between 8 and 12 weeks of age were used for these studies. Animals were group housed at the Laboratory Animal Services Unit (LASU) at the University of Saskatchewan (3 animals/cage) with a standard 12:12 light-dark cycle, *ad libitum* access to food and water, and environmental enrichment. Compounds were prepared in the vehicle [ethanol and cremophor in saline (1:1:8)] and administered intraperitoneally (i.p.). Catalepsy was assessed in the bar holding assay 5 min after compound administration with animals placed so that their forepaws clasped a 0.7-cm ring clamp 4.5 cm above the surface of the testing space (Garai et al., 2020). The length of time the ring was held was recorded up to 60 s (i.e., percent maximum possible effect [MPE] 60 s) with the trial ending if the mouse turned its head or body or made three consecutive escape attempts. Body temperature was measured 15 min after compound administration by using a rectal thermometer. Anti-nociceptive effects were measured in warm water (52 ± 2°C) using the tail-flick test 20 min after compound administration to a maximum of 20 s (i.e., percent maximum possible effect [MPE] 20 s). Compounds were administered at the doses indicated. Experimenters were blinded to treatment for all behavioral assessments and analyses. Animals were purchased, rather than bred, to reduce animal numbers. In all cases, experiments were performed with the approval of the University Animal Care Committee (UACC) at the University of Saskatchewan and in keeping with the guidelines of the Canadian Council on Animal Care (CCAC).

### Statistical analyses

[^3^H]CP55,940 binding data are represented as % change from maximal [^3^H]CP55,940 bound (i.e., 100%). Change in [^3^H]CP55,940 binding is represented as a downward deflection of the curve, and efficacy data are, therefore, represented as E_min_ (Table 1). HitHunter cAMP and PathHunter βarrestin2 data are shown as % of maximal CP55,940 response (i.e., 100%). Change in these assays is represented as an upward deflection of the curve, and efficacy data are, therefore, represented as E_max_ (Table 1). Concentration–response curves (CRCs) were fit using non-linear regression with variable slopes (four parameters) and used to calculate EC_50_, E_min_, and E_max_ (GraphPad, Prism, v. 9.0). Statistical analyses were conducted by one-way analysis of variance (ANOVA) using GraphPad. *Post hoc* analyses were performed using Tukey’s tests. Homogeneity of variance was confirmed using Bartlett’s test. All results are reported as the mean ± the standard error of the mean (SEM) or 95% confidence interval (CI), as indicated. *In vivo* data were analyzed via one-way ANOVA followed by Tukey’s *post hoc* test. GraphPad Prism 9.0 was used to analyze *in vivo* data, and *p* < 0.05 was considered to be statistically significant.

**TABLE 1:** Activity of compounds at hCB1R.

Compound	[^3^H]CP55,940		cAMP inhibition		βarrestin2 recruitment	
	K_i_ (nM)	E_min_ (%)	EC_50_ (nM)	E_max_ (%)	EC_50_ (nM)	E_max_ (%)
CP55,940	13 (5.6—33)	1.0 ± 4.7	1.0 (0.13—3.9)	100 ± 6.2	910 (700—1,200)	100 ± 3.3
∆^9^-THC	35 (17—71)	0.0 ± 3.5	5.2 (0.52—11)	70 ± 7.7*	600 (200—840)	47 ± 7.8*
∆^8^-THC	360 (120—1,000)*^#^	30 ± 5.0*^#^	440 (76—620)*^#^	56 ± 9.6*^#^	>10,000	21 ± 1.8*^#^
CBN	140 (47—670)*	71 ± 3.1*^#^	49 (14 – 160)*^#^	64 ± 4.6*^#^	>10,000	6.9 ± 0.96*^#^
∆^6a,10a^-THC	1,000 (470 – 2,000)*^#^	1.7 ± 6.5	600 (260—1,300)*^#^	100 ± 11^#^	>10,000	54 ± 4.9*
11-OH-∆^9^-THC	0.37 (0.10—1.3)*^#^	70 ± 1.7*^#^	11 (2.0—49)	28 ± 3.9*^#^	>10,000	47 ± 9.4*
Curcumin	>10,000	100 ± 2.0*^#^	>10,000	6.2 ± 0.76*^#^	>10,000	8.9 ± 2.2*^#^
Egc G	>10,000	95 ± 3.4*^#^	>10,000	5.6 ± 0.52*^#^	>10,000	7.2 ± 0.85*^#^
Olivetol	17 (3.3—90)	57 ± 5.5*^#^	>10,000	5.6 ± 1.6*^#^	>10,000	8.3 ± 2.1*^#^
PEA	>10,000	46 ± 5.3*^#^	730 (210—1,600)*^#^	26 ± 1.8*^#^	2,100 (920—3,100)	5.6 ± 3.3*^#^
Piperine	>10,000	37 ± 2.8*^#^	>10,000	5.5 ± 0.63*^#^	>10,000	7.9 ± 0.97*^#^
Quercetin	350 (44—860)*	78 ± 8.1*^#^	>10,000	4.6 ± 0.80*^#^	>10,000	7.0 ± 1.2*^#^
∆^8^-THC + ∆^9^-THC	2.6 (0.46—6.5)*^#^	7.0 ± 3.1	2.1 (0.58—12)	54 ± 1.6*^#^	>10,000	8.7 ± 3.0*^#^
CBN + ∆^9^-THC	170 (17—260)	20 ± 5.4	>10,000	60 ± 7.1*	>10,000	3.0 ± 0.21*^#^
∆^6a,10a^-THC + ∆^9^-THC	8.8 (0.29—14)^#^	1.5 ± 4.1	7.7 (0.89—19)	100 ± 4.4^#^	>10,000	56 ± 2.7*
11-OH-∆^9^-THC + ∆^9^-THC	1.2 (0.23—7.4)^#^	5.1 ± 3.1	11 (2.0—29)	23 ± 4.4*^#^	>10,000	44 ± 5.4*
Curcumin + ∆^9^-THC	>10,000	24 ± 6.8	3.1 (0.62—16)	2.4 ± 5.0*^#^	>10,000	1.8 ± 0.27*^#^
Egc G + ∆^9^-THC	7.9 (1.4—7.1)^#^	3.0 ± 3.9	4.0 (0.88—18)	13 ± 4.1*^#^	>10,000	2.5 ± 0.28*^#^
Olivetol + ∆^9^-THC	4.6 (0.83—29)	1.1 ± 2.7	>10,000	61 ± 7.7*	>10,000	1.4 ± 0.31*^#^
PEA + ∆^9^-THC	>10,000	1.7 ± 2.1	57 (10—280)	103 ± 4.8^#^	>10,000	14 ± 2.2*^#^
Piperine + ∆^9^-THC	>10,000	12 ± 7.2	>10,000	48 ± 7.6*^#^	>10,000	1.7 ± 0.28*^#^
Quercetin + ∆^9^-THC	350 (68—590)	3.2 ± 8.1	19 (5.3—95)	0.59 ± 4.3*^#^	>10,000	1.2 ± 0.44*^#^

Compound activity was quantified for [3H]CP55,940 binding, inhibition of forskolin-stimulated cAMP, or βarrestin2 recruitment in CHO cells stably expressing hCB1R and treated with phytomolecules. Data were fit to a variable slope (four parameters) non-linear regression in GraphPad (v. 9.0). *n* ≥ 6 independent experiments were performed in triplicate. E_max_ and E_min_ refer to the top and bottom of the concentration–response curves, respectively. Data are expressed as nM with 95% CI or %CP55,940 response, mean ± SEM. *p < 0.05 compared to CP55,940; #p < 0.05 compared to ∆^9^-THC within assay and measurement as determined via non-overlapping 95% CI or one-way ANOVA followed by Tukey’s *post hoc* test.

## Results

### Radioligand binding

**FIGURE 2:**
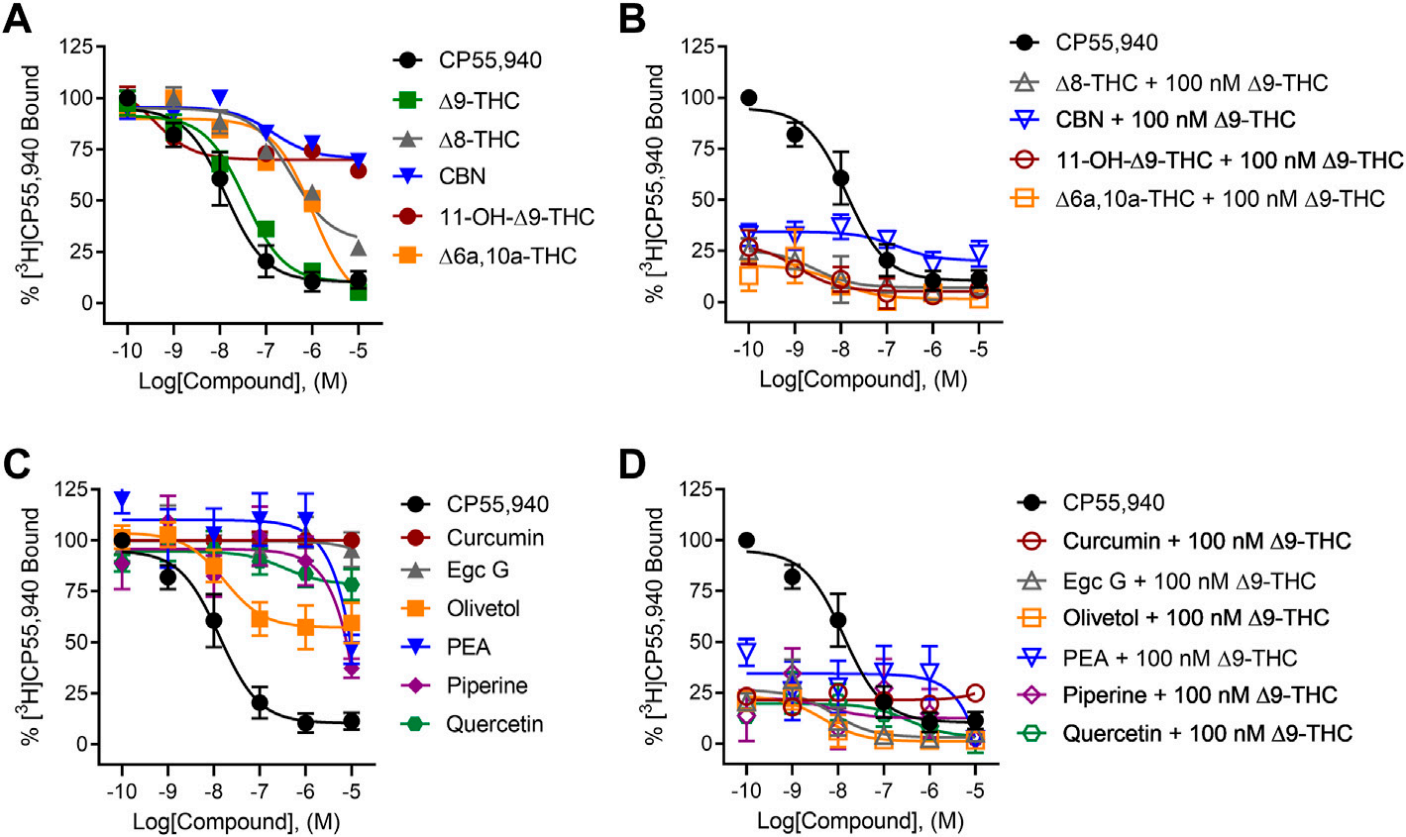
[^3^H]CP55,940 displacement from hCB1R CHO cell membranes. Compound activity was quantified for [^3^H]CP55,940 binding in CHO cells stably expressing hCB1R and treated with 0.1 nM—10 µM **(A)** cannabinoids; **(B)** cannabinoids + 100 nM ∆^9^-THC; **(C)** phytomolecules; and **(D)** phytomolecules + 100 nM ∆^9^-THC. Data were fit to a variable slope (four parameters) non-linear regression in GraphPad (v. 9). n ≥ 6 independent experiments were performed in duplicate. Data are expressed as mean ± SEM. K_i_ and E_min_ are reported in Table 1.

When tested alone, the cannabinoids (∆^8^-THC, ∆^6a,10a^-THC, 11-OH-∆^9^-THC, and CBN) reduced [^3^H]CP55,940 binding to hCB1R to some extent (Figure 2A). However, only ∆^6a,10a^-THC was able to fully compete [^3^H]CP55,940 from hCB1R (Figure 2A). The observed affinity (*K*_i_) for ∆^8^-THC, ∆^6a,10a^-THC, and 11-OH-∆^9^-THC was less than that of CP55,940 or ∆^9^-THC (Table 1). The affinity of CBN for hCB1R was less than that of CP55,940 but not different from ∆^9^-THC (Table 1). ∆^8^-THC, CBN, ∆^6a,10a^-THC, and 11-OH-∆^9^-THC displayed greater *E*_min_ values than either CP55,940 or ∆^9^-THC, indicating that they did not completely displace [^3^H]CP55,940 from hCB1R and suggesting an incomplete overlap of binding sites between these compounds and [^3^H]CP55,940 (Table 1). These cannabinoids were further assessed in the presence of 100 nM ∆^9^-THC to determine whether these cannabinoids altered the binding of ∆^9^-THC to hCB1R (Figure 2B). Each of these cannabinoids augmented the displacement of [^3^H]CP55,940 from hCB1R by 100 nM ∆^9^-THC relative to 100 nM ∆^9^-THC alone (∼20%; Figure 2B). ∆^8^-THC and ∆^6a,10a^-THC displayed lower *K*_i_ values when co-administered with 100 nM ∆^9^-THC for hCB1R relative to being administered alone, suggesting some form of cooperativity between these compounds. No change in *K*_i_ was observed for 11-OH-∆^9^-THC + 100 nM ∆^9^-THC or CBN + 100 nM ∆^9^-THC compared to these compounds alone, suggesting that 11-OH-∆^9^-THC and CBN were primarily responsible for [^3^H]CP55,940 displacement in the presence of 100 nM ∆^9^-THC (Figure 2B; Table 1). These data indicate that select cannabinoids were able to displace [^3^H]CP55,940 from hCB1R either alone or in concert with ∆^9^-THC. For ∆^8^-THC and ∆^6a,10a^-THC, these ligands may have engaged hCB1R cooperatively to displace [^3^H]CP55,940, but this is not a reflection of functional signaling outcomes at hCB1R.

In contrast to cannabinoids, when the other phytomolecules were tested alone, only olivetol produced a clear reduction in [^3^H]CP55,940 binding to hCB1R with an estimated *K*_i_ that was not different from CP55,940 or ∆^9^-THC but did not completely displace [^3^H]CP55,940 from hCB1R, suggesting that the displacement was not competitive (Figure 2C). Other phytomolecules tested produced some minimal degree of [^3^H]CP55,940 displacement at the highest concentrations tested (i.e., 1 and 10 μM) (Figure 2C; Table 1). These phytomolecules were further assessed in the presence of 100 nM ∆^9^-THC to determine whether they altered the binding of ∆^9^-THC to hCB1R (Figure 2D). Curcumin, olivetol, PEA, piperine, and quercetin did not significantly alter [^3^H]CP55,940 displacement from hCB1R in the presence of 100 nM ∆^9^-THC (Table 1). Egc G augmented the displacement of [^3^H]CP55,940 from hCB1R by ∆^9^-THC as indicated by the decrease in observed *K*_i_ relative to ∆^9^-THC alone (Table 1).

### Inhibition of forskolin-stimulated cAMP

**FIGURE 3:**
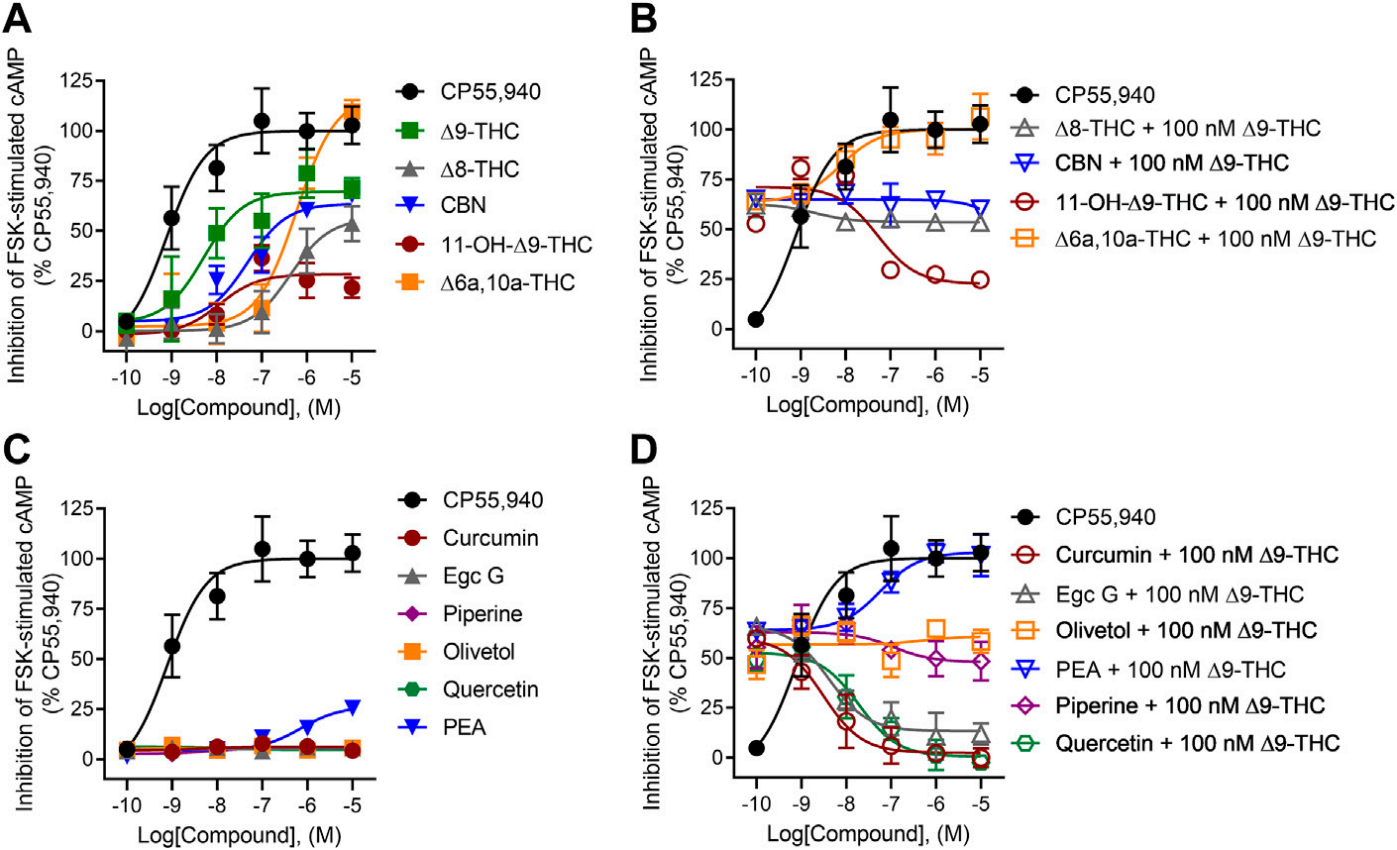
hCB1R-dependent inhibition of forskolin-stimulated cAMP accumulation. Compound activity was quantified for inhibition of 10 µM forskolin-stimulated cAMP accumulation in CHO cells stably expressing hCB1R and treated with 0.1 nM—10 µM **(A)** cannabinoids; **(B)** cannabinoids + 100 nM ∆^9^-THC; **(C)** phytomolecules; and **(D)** phytomolecules + 100 nM ∆^9^-THC for 90 min according to the standard operating procedures of the HitHunter assay. Data were fit to a variable slope (four parameters) non-linear regression in GraphPad (v. 9). n ≥ 6 independent experiments were performed in triplicate. Data are expressed as mean ± SEM. EC_50_ and E_max_ are reported in Table 1.

All tested cannabinoids increased hCB1R-mediated inhibition of cAMP accumulation (Figure 3A). All cannabinoids tested were partial agonists of cAMP inhibition except for ∆^6a,10a^-THC, which was a full agonist with low potency (Figure 3A). ∆^8^-THC, CBN, and ∆^6a,10a^-THC were approximately 440x, 49x, and 600x less potent, respectively, than C55,940, whereas the ∆^9^-THC metabolite, 11-OH-∆^9^-THC, was not statistically different in its potency compared to CP55,940 and ∆^9^-THC (Table 1). Similarly, ∆^8^-THC, CBN, and 11-OH-∆^9^-THC displayed lower efficacy than CP55,940 and ∆^9^-THC, consistent with previous observations that all of these compounds are hCB1R partial agonists (Table 1) (Ross et al., 1999; ElSohly et al., 2017). In the presence of 100 nM ∆^9^-THC, neither ∆^8^-THC nor CBN produced a significant change in cAMP inhibition relative to 100 nM ∆^9^-THC (Figure 3B), likely due to their weak hCB1R affinity as observed in [^3^H]CP55,940 competition binding experiments. Also, 11-OH-∆^9^-THC inhibited the activity of 100 nM ∆^9^-THC in a concentration-dependent manner and to the level of 11-OH-∆^9^-THC agonism consistent with this ligand’s competition for hCB1R binding and activation (Figure 3B). ∆^6a,10a^-THC elevated cAMP inhibition above the activity of 100 nM ∆^9^-THC in a concentration-dependent manner and to the level of ∆^6a,10a^-THC agonism consistent with that ligand’s higher efficacy at hCB1R, but with greater potency than ∆^6a,10a^-THC alone (Figure 3B; Table 1). This observation with ∆^6a,10a^-THC may suggest that the compound is able to engage hCB1R cooperatively with ∆^9^-THC to facilitate G-protein-dependent signaling, consistent with the observations made with [^3^H]CP55,940. Whether this *in vitro* observation has a direct outcome *in vivo* is not clear from these data.

Among the other phytomolecules tested, only PEA produced weak partial agonist inhibition of cAMP accumulation, having potency and efficacy significantly lower than either CP55,940 or ∆^9^-THC (Figure 3C; Table 1). Olivetol and piperine did not change the cAMP inhibitory effects of 100 nM ∆^9^-THC, confirming that although these compounds produced some displacement of [^3^H]CP55,940, they are not agonists of hCB1R (Figure 3D; Table 1). Curcumin, Egc G, and quercetin inhibited the activity of 100 nM ∆^9^-THC in a concentration-dependent manner inconsistent with these ligands’ weak hCB1R affinity in [^3^H]CP55,940 binding experiments (Figure 3D; Table 1). Therefore, curcumin, Egc G, and quercetin likely inhibited ∆^9^-THC-dependent cAMP inhibition via an indirect mechanism such as altering membrane dynamics, G-protein coupling, or adenylate cyclase activity (Jonsson et al., 2001; Balasubramanyam et al., 2004; Ho et al., 2008; Choi et al., 2009). If such *in vitro* observations manifested *in vivo*, then curcumin, Egc G, and quercetin could limit the effects of ∆^9^-THC, such as intoxication. Lastly, PEA augmented the activity of 100 nM ∆^9^-THC in a concentration-dependent manner inconsistent with PEA’s weak hCB1R affinity in [^3^H]CP55,940 binding experiments (Figure 3D; Table 1). PEA has been described as a partial hCB1R agonist and bears structural similarity to the endogenous cannabinoid anandamide (AEA) (Jonsson et al., 2001; Ho et al., 2008). Therefore, PEA’s activity may be consistent with allosteric agonism of hCB1R (Laprairie et al., 2019; Garai et al., 2020, 2021).

### βarrestin2 recruitment

**FIGURE 4:**
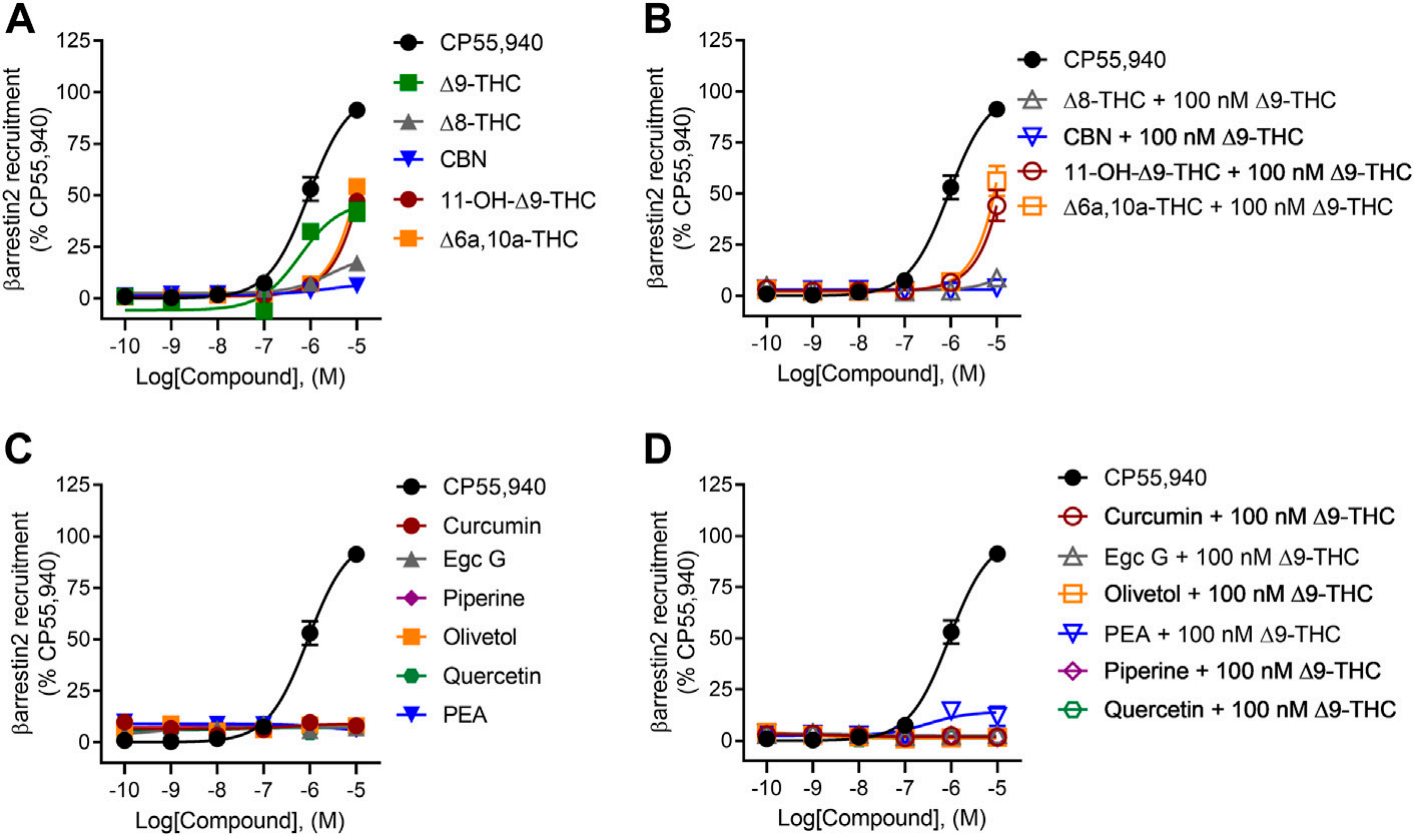
hCB1R-dependent recruitment of βarrestin2. Compound activity was quantified for βarrestin2 recruitment in CHO cells stably expressing hCB1R and treated with 0.1 nM—10 μM **(A)** cannabinoids; **(B)** cannabinoids + 100 nM ∆^9^-THC; **(C)** phytomolecules; and **(D)** phytomolecules+ 100 nM ∆^9^-THC for 90 min according to the standard operating procedures of the PathHunter assay. Data were fit to a variable slope (four parameters) non-linear regression in GraphPad (v. 9). n ≥ 6 independent experiments were performed in triplicate. Data are expressed as mean ± SEM. EC_50_ and E_max_ are reported in Table 1.

All tested cannabinoids were weak partial agonists of βarrestin2 recruitment, although only ∆^9^-THC produced quantifiable potency within the concentration range used (Figure 4A; Table 1). The maximum efficacy observed for ∆^8^-THC and CBN was lower than that of CP55,940 and ∆^9^-THC (Figure 4A; Table 1). Given the minimal activity observed in the βarrestin2 recruitment assay, it is not surprising that the cannabinoids did not drastically alter βarrestin2 recruitment in the presence of 100 nM ∆^9^-THC (Figure 4B). At the highest concentration tested, 10 μM 11-OH-∆^9^-THC and ∆^6a,10a^-THC elevated βarrestin2 recruitment above the activity of 100 nM ∆^9^-THC in a concentration-dependent manner and to the level of their agonism consistent with those ligands’ efficacy at hCB1R (Figure 4B).

None of the phytomolecules tested promoted βarrestin2 recruitment when tested alone (Figure 4C). Only one of the phytomolecules tested—PEA—altered the recruitment of βarrestin2 by 100 nM ∆^9^-THC (Figure 4D; Table 1). Similar to its activity in the cAMP inhibition assay, PEA slightly increased βarrestin2 recruitment in the presence of 100 nM ∆^9^-THC, consistent with the notion that PEA may be an allosteric agonist of hCB1R (Figure 4D; Table 1).

### Confirming hCB1R dependence of PEA-mediated cAMP inhibition

**FIGURE 5:**
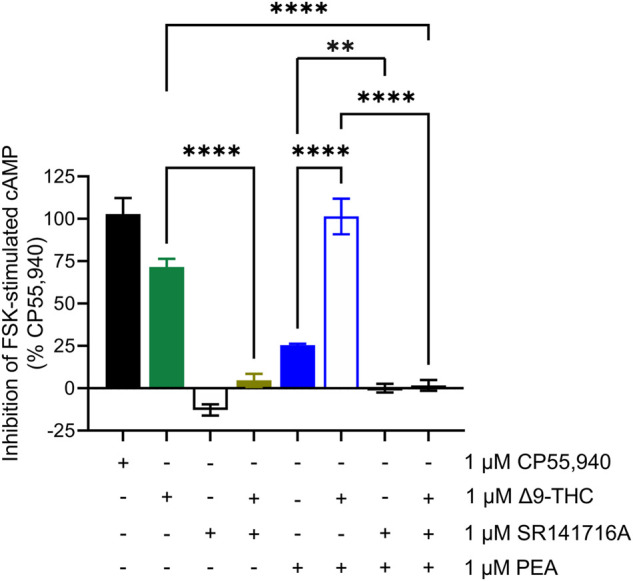
hCB1R-dependent inhibition of forskolin-stimulated cAMP accumulation. Compound activity was quantified for inhibition of 10 μM forskolin-stimulated cAMP accumulation in CHO cells stably expressing hCB1R and treated with compounds as indicated for 90 min according to the standard operating procedures of the HitHunter assay. Data were analyzed in GraphPad (v. 9). *n* = 6 independent experiments were performed in triplicate. Data are expressed as mean ± SEM. **p < 0.01, ****p < 0.0001 as determined by one-way ANOVA followed by Tukey’s *post hoc* test for multiple comparisons.

In general, we observed partial agonism by the tested cannabinoids and an absence of hCB1R-dependent activity by the other phytomolecules tested. One exception to this generalization was PEA. PEA augmented hCB1R-dependent cAMP inhibition and βarrestin2 recruitment with ∆^9^-THC but displayed little to no affinity for the orthosteric site of hCB1R as observed by radioligand binding experiments. The ability of PEA to activate hCB1R was tested in the cAMP inhibition assay in the absence or presence of SR141716A as an hCB1R antagonist/inverse agonist (Figure 5). Co-treatment of hCB1R CHO cells with 1 μM PEA and 1 μM SR141716A—or a combination of 1 μM PEA, 1 μM ∆^9^-THC, and 1 μM SR141716A—completely prevented PEA-dependent cAMP inhibition (Figure 5). Previous reports have shown that allosteric or indirect agonism of hCB1R is blocked by SR141716A even when those allosteric ligands are not displaced in radioligand binding experiments (Garai et al., 2020, Garai et al., 2021). Therefore, the collective evidence supports PEA indirectly augmenting the activity of hCB1R *in vitro*.

### *In vivo* evaluation

Given our *in vitro* observations, we wanted to determine whether minor cannabinoids and combinations of ∆^9^-THC with select phytomolecules affected catalepsy, body temperature, and nociception in mice, which are three physiological parameters well known to be affected by ∆^9^-THC. Specifically, we chose to examine whether 10 mg/kg PEA, curcumin, or quercetin altered physiological responses to 10 mg/kg ∆^9^-THC because these phytomolecules had augmented (PEA) or inhibited (curcumin and quercetin) ∆^9^-THC-dependent inhibition of cAMP to the greatest extent. A dose of 10 mg/kg was chosen because it has been used previously to demonstrate ∆^9^-THC-dependent intoxicating effects in rodent models by ourselves and others; and ∆^9^-THC plasma levels similar to intoxicating levels were observed after acute cannabis use in humans (Craft and Leitl, 2008; Wakley et al., 2014; Grim et al., 2017; Nguyen et al., 2018; Moore et al., 2021).

**FIGURE 6:**
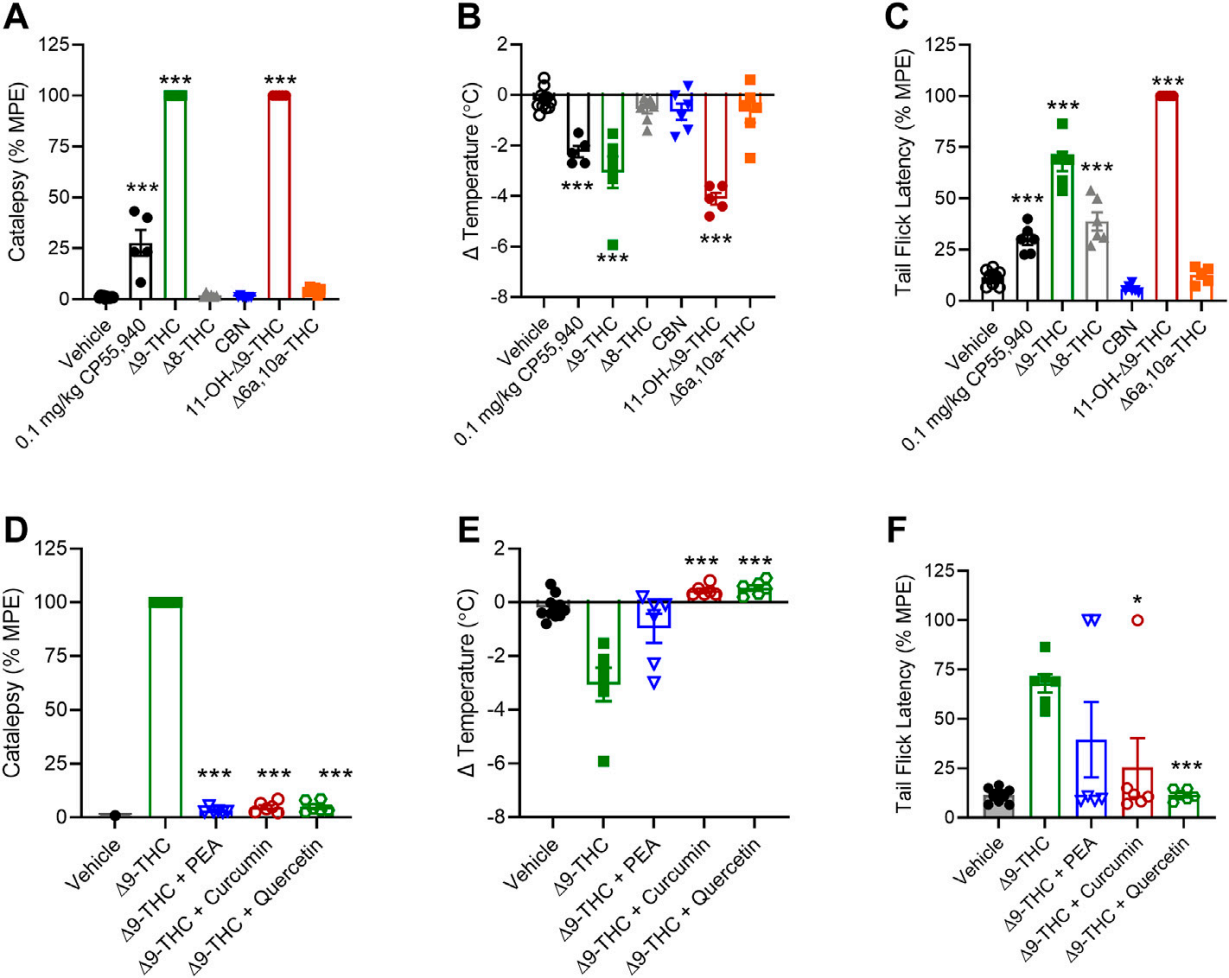
*In vivo* effects of cannabinoids and select phytomolecules in male C57BL/6 mice. Male mice were treated with 10 mg/kg cannabinoids **(A–C)** or 10 mg/kg ∆^9^-THC + 10 mg/kg select phytomolecules i. p. **(D–F)** and assessed for catalepsy 5 min post-injection **(A,D)**, body temperature 15 min post-injection **(B,E)**, and nociception in the tail-flick assay 20 min post-injection **(C,F)**. Data for catalepsy are represented as % MPE during a maximum 60 s trial. Data for the tail-flick assay are represented as % MPE during a maximum 20 s trial. All data were analyzed in GraphPad (v. 9). *n* = 5–10 animals per treatment group. Data are expressed as mean ± SEM. *p < 0.05, **p < 0.01, and ****p < 0.001 as determined by one-way ANOVA followed by Tukey’s *post hoc* test for multiple comparisons.

Among the cannabinoids tested, ∆^8^-THC, CBN, and ∆^6a,10a^-THC did not produce catalepsy or reduce body temperature in male mice (Figures 6A,B). ∆^8^-THC produced an anti-nociceptive effect that was not different from ∆^9^-THC, but CBN and ∆^6a,10a^-THC did not produce such effect (Figure 6C). Also, 11-OH-∆^9^-THC produced equal catalepsy and greater hypothermia and anti-nociceptive responses relative to ∆^9^-THC (Figures 6A–C).

When 10 mg/kg PEA, curcumin, or quercetin was tested in combination with 10 mg/kg ∆^9^-THC, each of these phytomolecules was capable of blocking ∆^9^-THC’s effects on catalepsy and body temperature (Figures 6D,E). Curcumin and quercetin also blocked ∆^9^-THC’s effect in the nociception test, but PEA did not block the effect (Figure 6F). In the case of PEA, the mechanism underlying the observation is unclear because *in vitro* PEA had augmented ∆^9^-THC’s effects. PEA may have multiple intracellular targets including peroxisome proliferator-activated receptor *α* (PPARα), GPR55, and GPR119 (Lo Verme et al., 2005; Godlewski et al., 2009). Our results and earlier studies also indicate PEA is unlikely to be a CB1R ligand (O’Sullivan and Kendall, 2010). Therefore, the observed *in vivo* effects of PEA here may be due to PEA acting on other ligand targets not present in our *in vitro* model.

## Discussion

In general, we observed that all cannabinoids tested—∆^9^-THC, ∆^8^-THC, ∆^6a,10a^-THC, 11-OH-∆^9^-THC, and CBN—behaved as CB1R partial agonists in the cAMP inhibition assay and were able to partially displace [^3^H]CP55,940 from CB1R in the competition binding assay. Together, these data support the hypothesis that the cannabinoids tested here are all weak and/or partial agonists of CB1R. These data are in agreement with previously published findings on ∆^8^-THC, CBN, and 11-OH-∆^9^-THC (Ross et al., 1999; Pertwee, 2008), and this study represents the first report of activity for ∆^6a,10a^-THC. The incomplete competition of ∆^8^-THC, CBN, and 11-OH-∆^9^-THC with [^3^H]CP55,940 suggests that the occupied binding site of these ligands differs slightly from that of CP55,940. It is possible that only a subset of amino acids in the CB1R ligand-binding site is engaged by these cannabinoids compared to CP55,940, as shown for CBD, Org27569, rimonabant, and anandamide (Iliff et al., 2011; Baillie et al., 2013; Laprairie et al., 2015; Hua et al., 2017). Future experiments utilizing site-directed mutagenesis are needed to assess this question directly.

After the most abundant phytocannabinoid ∆^9^-THC, ∆^8^-THC, ∆^6a,10a^-THC, and CBN are the phytocannabinoids that are most commonly found as less abundant THC-like isoforms in *Cannabis* products (Pertwee, 2008; ElSohly and Gul, 2014; Howlett and Abood, 2017). Previous work has shown that ∆^8^-THC behaves as a weak partial agonist of CB1R in GTP binding experiments, consistent with our observations (Ross et al., 1999; Pertwee, 2008). Comparatively, little is known about ∆^6a,10a^-THC; however, its structural similarities to ∆^9^-THC suggest that it is likely to have some cannabinoid receptor modulatory activity (Howlett and Abood, 2017). CBN is both a minor phytocannabinoid and an oxidation product of ∆^9^-THC found to accumulate in *Cannabis* products during storage (Felder et al., 1995; Showalter et al., 1996; Howlett and Abood, 2017). Our data suggesting that CBN is a weak partial CB1R agonist are in agreement with previous findings for CBN for both relative potency and efficacy in CHO and AtT-20 cells expressing human CB1R and *ex vivo* tissue studies (Felder et al., 1995; Showalter et al., 1996). The ∆^9^-THC metabolite 11-OH-∆^9^-THC has previously been shown to produce CB1R-dependent effects in the rodent tetrad model; our data support earlier work showing CB1R activity for this ligand (Huestis, 2005). The potency and efficacy of 11-OH-∆^9^-THC approximate data observed in earlier works (Felder et al., 1995; Showalter et al., 1996; Huestis, 2005). The weak partial agonist effects displayed by these ligands suggest that they may in fact be functionally antagonistic in the presence of higher agonist concentrations and *in vivo*. This functional antagonism has been previously demonstrated for ∆^9^-THC itself (Laprairie et al., 2015; Grim et al., 2017). Future work assessing the potential antagonist activity of these compounds in the presence of a full agonist such as CP55,940 will be able to better classify the mechanisms of action for these compounds beyond what has been performed here.

Comparing our *in vitro* data with our *in vivo* observations suggests that although cannabinoids may be capable of activating CB1R-dependent pathways *in vitro*, these observations may not translate to *in vivo* effects. Of note, however, 11-OH-∆^9^-THC displayed the greatest estimated binding affinity to CB1R, greatest potency in the cAMP inhibition assay among minor phytocannabinoids, and similar efficacy to ∆^9^-THC in the βarrestin2 recruitment assay and proved to have the greatest *in vivo* activity. Additional work is required to determine other pharmacological targets of the minor cannabinoids *in vivo* beyond CB1R.

Among the non-cannabinoid phytomolecules tested here, we observed generally weak, minimal, and partial displacement of [^3^H]CP55,940 in competition binding assays. One notable exception to this was olivetol, which produced a concentration-dependent reduction in [^3^H]CP55,940 binding. It is unclear whether this loss of [^3^H]CP55,940 binding was a consequence of direct competition or indirect changes in membrane integrity and lipid dynamics that may occur with these highly lipophilic ligands (Howlett and Abood, 2017). Of note, olivetol did not significantly impact CB1R-dependent signal transduction in other assays. Additional data are required to assess the specific mechanism of competition occurring for olivetol in these experiments. PEA only reduced [^3^H]CP55,940 binding at concentrations exceeding 1 μM but augmented CB1R- and ∆^9^-THC-dependent inhibition of cAMP and βarrestin2 recruitment at approximately 1 μM. These observations for PEA are consistent with a partial agonist or positive allosteric modulator (Jonsson et al., 2001; Ho et al., 2008). PEA is structurally similar to both endogenous cannabinoids anandamide and 2-arachidonoylglycerol, and given this structural similarity, it would not be surprising if the compound activated CB1R. Additional experiments are required to assess whether these effects are allosteric in nature, as indirectly assessed by others (Jonsson et al., 2001; Ho et al., 2008). Finally, the three polyphenolic terpenoid compounds—quercetin, Egc G, and curcumin—all displayed minimal competition with [^3^H]CP55,940 at CB1R but surprisingly produced a concentration-dependent and potent antagonistic inhibition of CB1R- and ∆^9^-THC-mediated inhibition of cAMP. Given these data, we propose that the polyphenolic terpenes tested here indirectly inhibit signaling downstream of CB1R. Quercetin, Egc G, and curcumin have all been shown to have widespread and non-specific antioxidant, lipid raft, and transcription factor modulatory effects that could interfere with GPCR-dependent signaling and trafficking (Balasubramanyam et al., 2004; Choi et al., 2009). When exploring a subset of these phytomolecules *in vivo*, curcumin’s and quercetin’s effects are congruent with *in vitro* observations where both phytomolecules inhibited ∆^9^-THC-dependent catalepsy, hypothermia, and anti-nociception.

The limitation of this work is that it is an assessment of the pharmacology for a subset of *Cannabis*- and non-*Cannabis-*derived phytomolecules at a single cannabinoid receptor, CB1R. Importantly, this study also focused only on male mice. Planned future studies will incorporate female mice as we and others have observed cannabinoid-dependent sex differences *in vivo* (Craft and Leitl, 2008; Wakley et al., 2014; Kim et al., 2022). Although caution should be exercised when extrapolating *in vitro* cell culture data into the whole animal *in vivo* systems, three observations from our dataset warrant further consideration with regard to effecting cannabis pharmacology. First, the majority of compounds tested—exceptions being curcumin and Egc G—partially or fully competed with [^3^H]CP55,940 for receptor binding. Therefore, it is possible that these phytomolecules could reduce ∆^9^-THC binding at CB1R and thus diminish its medicinal and intoxicating effects if given at least at equimolar concentrations. In particular, ∆^8^-THC, ∆^6a,10a^-THC, and olivetol, with their more potent and near complete competition of CP55,940, could limit ∆^9^-THC’s binding to CB1R. Second, the cannabinoids tested all yielded CB1R-dependent inhibition of cAMP with less potency and efficacy than ∆^9^-THC, with the exception of ∆^6a,10a^-THC which displayed greater efficacy. When administered alone, we observed that 11-OH-∆^9^-THC was able to reproduce or exceed the effects of ∆^9^-THC *in vivo*. If, however, both ∆^9^-THC and the cannabinoids tested were present together, it is unlikely that their actions would be additive or synergistic as these effects appear to be CB1R-dependent. In contrast, two compounds—curcumin and quercetin—were able to diminish the intoxicating effects of ∆^9^-THC via indirect inhibition of CB1R-dependent signaling. Third, two cannabinoids—∆^6a,10a^-THC and 11-OH-∆^9^-THC—produced βarrestin2 recruitment similar to that of ∆^9^-THC. At other GPCRs, such as the μ-opioid and serotonin 2A receptors, βarrestin2 recruitment is associated with the intoxicating and impairing effects of receptor activation (Bohn et al., 1999; Schmid et al., 2017; Wacker et al., 2017). Given 11-OH-∆^9^-THC’s *in vivo* activity observed here, it is possible that this compound specifically could yield intoxicating effects akin to ∆^9^-THC via βarrestin2 (Felder et al., 1995; Showalter et al., 1996; Huestis, 2005), whereas the other compounds tested here would not yield βarrestin2-dependent intoxicating effects.

Although changes in [^3^H]CP55,940 binding were observed for several cannabinoids and phytomolecules, it is not presently clear whether changes in signaling or protein recruitment were the result of direct, orthosteric activity as opposed to allosteric modulation, or modulation of a separate target whose signaling converges on the same output measure (e.g., membrane fluidity and adenylate cyclase, etc.). In the past, our group and others have explored cannabinoid allostery (Laprairie et al., 2015; Martinez-Pinilla et al., 2017; Tham et al., 2018; Navarro et al., 2021), but this was not the focus of the present study. ∆^9^-THC and other cannabinoids affect signaling via many other proteins, including the type 2 cannabinoid receptor (CB2R), the orphan GPCR GPR55, 5HT1A receptor, μ-opioid receptor, peroxisome proliferator-activated receptors (PPARs), and the transient receptor potential vanilloid 1 Ca^2+^ channel (TRPV1), among others (Howlett and Abood, 2017). Therefore, in order to understand the poly-pharmacology of cannabinoids and terpenes in whole organisms, other receptor targets and pharmacokinetic outcomes must be considered.

In conclusion, there exists a great wealth and array of phytomolecules present in cannabis whose pharmacology as single chemicals is still being determined, let alone as a milieu of more than five hundred compounds. Our studies are the first steps toward characterizing the complex mixture of compounds present in cannabis as well as their effects and interactions. It is our goal and the goal of many researchers exploring the potential of an entourage effect (Santiago et al., 2019; Devsi et al., 2020; Finlay et al., 2020; Heblinski et al., 2020; LaVigne et al., 2021) and to determine if and how cannabinoids and terpenes may influence one another’s pharmacology. As interest continues to accrue with respect to the use of cannabis for medical and non-medical purposes, the aim of this work was to understand how cannabis and non-cannabis phytomolecules work alone and in combination with one another so they can be used safely.

## Data Availability

The original contributions presented in the study are included in the article/Supplementary Material; further inquiries can be directed to the corresponding author.
